# Pharmacogenetics and Molecular Ancestry of *SLC22A1*, *SLC22A2*, *SLC22A3*, *ABCB1*, *CYP2C8*, *CYP2C9*, and *CYP2C19* in Ecuadorian Subjects with Type 2 Diabetes Mellitus

**DOI:** 10.3390/ph18091335

**Published:** 2025-09-05

**Authors:** Adiel Ortega-Ayala, Carla González de la Cruz, Lorena Mora, Mauro Bonilla, Leandro Tana, Fernanda Rodrigues-Soares, Pedro Dorado, Adrián LLerena, Enrique Terán

**Affiliations:** 1 Department of Pharmacology, Faculty of Medicine, National Autonomous University of Mexico, Mexico City 04510, Mexico; ad.ortega@unam.mx; 2 Personalised Medicine and Mental Health Unit, University Institute for Biosanitary Research of Extremadura (INUBE), 06080 Badajoz, Spain; carla.gonzalezd@externos.salud-juntaex.es (C.G.d.l.C.); fernanda.soares@uftm.edu.br (F.R.-S.); pdorado@unex.es (P.D.); 3 Pharmacogenomics and Personalized Medicine Unit, Badajoz University Hospital, Extremadura Health Service SES, 06006 Badajoz, Spain; 4 RIBEF Red Iberoamericana de Farmacogenégica y Farmacogenómica SIFF, 06080 Badajoz, Spain; 5 Laboratorio Clínico, Dispensario Central del Instituto Ecuatoriano de Seguridad Social (IESS), Quito 170901, Ecuador; loremorad@yahoo.es; 6 Colegio de Ciencias de la Salud, Universidad San Francisco de Quito (USFQ), Quito 170901, Ecuador; mauro.bonilla95@gmail.com (M.B.); leandro.tana@gmail.com (L.T.); 7 Department of Pathology, Genetic and Evolution, Universidade Federal do Triângulo Mineiro, Uberaba 38025-350, Brazil

**Keywords:** antidiabetic drugs, T2DM, *CYP2C8*, *CYP2C9*, *CYP2C19*, molecular ancestry, SLC22 family

## Abstract

**Background/Objectives:** In Ecuador, the prevalence of type 2 diabetes mellitus (T2DM) is the second leading cause of death after ischemic heart disease. Genetic variability in protein-coding genes, single nucleotide variants (SNVs), influences the response to antidiabetic drugs. The frequency of SNVs varies among different populations, so studying the ancestral proportions among SNVs is important for personalized medicine in the treatment of T2DM. This study aimed to evaluate the distribution of Native American, European, and African (NATAM, EUR, and AFR) ancestry in 23 allelic variants of the seven genes that encode the relevant enzymes that metabolize antidiabetic drugs in an Ecuadorian population. **Methods:** Twenty-three allelic variants of seven genes were analyzed in 297 patients with T2DM from Ecuador, and the molecular ancestry of the samples was analyzed considering three ancestral groups, NATAM, EUR, and AFR using 90 ancestry informative markers (AIMs). Allele and ancestry distributions were analyzed using Spearman’s correlation. **Results**: The Ecuadorian population presents NATAM (61.33%), EUR (34.48%), and AFR (2.60%) ancestry components. CYP2C8*1 and CYP2C9*1 were positively related to NATAM ancestry, while CYP2C8*4 and CYP2C9*2 were positively related to EUR ancestry. CYP2C19*17 was positively correlated to AFR ancestry. The correlation of *SLC22A1* variants such as A in rs594709 was positively correlated with NATAM, while GAT in rs72552763 was positive for EUR. The G variant of rs628031 of the *SLC22A1* gene was positively correlated with NATAM and negatively correlated with EUR. The C variant of rs2076828 of the *SLC22A3* gene was positively correlated with NATAM ancestry. **Conclusions:** In the Ecuadorian population, a predominance of Native American ancestry has been observed. Among the allelic variants related to enzymes that metabolize antidiabetic drugs, a relationship has been observed between this ancestral component and variants of the CYP2C8*1, CYP2C9*1, *SLC22A1* (rs594709 and rs628031)*,* and *SLC22A3* (rs2076828) genes. This information is fundamental for the development of strategies for the implementation of personalized medicine programs for Latin American patients.

## 1. Introduction

Type 2 diabetes mellitus (T2DM) accounts for a major public health problem worldwide due to its high prevalence, increasing incidence, high morbidity and mortality rates, and considerable healthcare costs. T2DM currently affects more than 500 million people, and the healthcare burden is unevenly distributed, disproportionately affecting low- and middle-income countries, which account for approximately 80% of the global diabetic population [1].

According to the International Diabetes Federation, the highest rates of T2DM incidence in young populations are found among Canadian First Nations, Native Americans, Indigenous Australians, and Afro-Latin Americans. In contrast, the lowest incidence rates are found among Europeans and white Americans [2]. These differences are attributed to interethnic variability, genetic predisposition, and disparities in access to healthcare [2].

It is estimated that approximately 40% of people with T2DM in Latin America are undiagnosed, making it difficult to accurately estimate the costs to healthcare systems of treating and caring for patients with T2DM [3].

In Ecuador, the prevalence of diabetes is 7.1% [4], and it is the second leading cause of death after ischemic heart disease, for which T2DM is also a risk factor [5].

Metformin remains the first-line pharmacological treatment for T2DM. However, when therapeutic goals are not achieved with monotherapy, combination therapy is recommended. The most effective agents used in combination include pioglitazone, sodium-glucose cotransporter 2 inhibitors (SGLT2i), glucagon-like peptide-1 receptor agonists (GLP-1), and sulfonylureas (SU) [6].

Despite the wide range of pharmacological treatments available, interindividual variability in response to drugs remains a significant challenge. This variability is largely influenced by variations in the genes involved in the pharmacokinetics and pharmacodynamics of antidiabetic drugs, accounting for 20–30% of these differences in pharmacological response [7].

Genetic variation not only affects therapeutic efficacy but can also contribute to the occurrence of adverse drug reactions (ADRs). Specifically, 59% of drugs commonly associated with ADRs are metabolized by enzymes encoded by allelic variants with altered function [8]. This is important, as it has now been shown that overall mortality due to adverse events has tripled between 2001 and 2019 [9]. Specifically, hypoglycemic agents are one of the drugs of greatest concern in the occurrence of ADRs [10].

The mechanism of action of metformin is mainly through the inhibition of hepatic glucose production, and its cellular uptake and distribution depend on organic cation transporters (OCTs), particularly OCT1, OCT2, OCT3, and P-glycoprotein (P-gp). These transporters are encoded by highly polymorphic genes *SLC22A1*, *SLC22A2*, *SLC22A3*, and *ABCB1*, respectively [11], which are associated with the therapeutic response to metformin and play a fundamental role in its pharmacokinetics [12]. An association has been demonstrated between genetic variants of *SLC22A1* and the onset of gastrointestinal ADRs in diabetic patients treated with metformin [13,14].

Drugs such as SU and thiazolidinediones (TZD) are metabolized mainly by cytochrome P450 (CYP450) enzymes. Specifically, SUs are mainly metabolized by CYP2C9 and CYP2C19 [15], while TZDs are metabolized by CYP2C8 [16]. Several studies have described an association between reduced CYP2C9 function and an increased risk of hypoglycemia secondary to SU use [17,18]. In the case of TZDs, there are studies that report the appearance of edema or lower weight gain in patients carrying the CYP2C8*3 genetic variant compared to wild-type patients [19]. Therefore, in managing the disease, the goal is to identify the most appropriate treatment for each patient, for which it is essential to incorporate the analysis of pharmacogenetic biomarkers into clinical practice, both for disease prediction and for pharmacological monitoring [20].

In sum, seven genes have been described as the most relevant for the pharmacogenetics of antidiabetic drugs: *CYP2C8* for TZDs, *CYP2C9* and *CYP2C19* for SU, and *SLC22A1, SLC22A2*, *SLC22A3*, and *ABCB1* for metformin.

An association has been demonstrated between genetic variants relevant to drug response and the ethnogeographic distribution of the population. Therefore, information on the distribution of these variants is important for guiding specific therapies for each population [21,22]. However, to date, no pharmacogenetic studies have specifically addressed the influence of the unique genetic ancestry of the Ecuadorian population diagnosed with T2DM. Most pharmacogenetic investigations aiming to optimize antidiabetic drug prescription have been conducted predominantly in European populations, potentially limiting the applicability of their findings to the Ecuadorian context [23]. This fact is especially relevant in populations with weak health systems, which are often accompanied by a shortage of medicines for the treatment of relevant diseases such as T2DM [24].

As in other populations in South America, the Ecuadorian population is multicultural and multiethnic, resulting from multiple migratory events and admixture over more than 525 years. This means that this population is characterized by the continuous mixing of European, Amerindian, and African individuals, giving rise to great ethnic diversity [25]. At present, Ecuador comprises three main ethnic groups: the mestizo population, Native Amerindian, and Afro-Ecuadorian. The mestizo population represents approximately 85% of the total population and is a mixed group of European and Amerindian descent (NATAM) [25].

The study of population-specific genetic variants, with the aim of providing evidence to optimize the clinical implementation of pharmacogenetics in Latin American populations, has been the ultimate goal of the RIBEF (Ibero-American Network of Pharmacogenetics and Pharmacogenomics). This has involved initial studies of self-reported ancestry in individuals [26], followed by subsequent analyses of the molecular ancestry of the population [27]. The main objective is to develop strategies for the implementation of pharmacogenetics adapted to the Latin American population [21,22]. In a previous study in Mexico [28,29], the RIBEF studies were initiated in diabetic patients, one of the most prevalent diseases in this region that causes relevant health problems, finding relevant information regarding the relevance of the ancestral component, pharmacogenetic polymorphisms and the response to antidiabetic drug treatment. In this Mexican DMT2 study, the percentage of NATAM ancestry was 65.48% and was positively correlated with CYP2C8*1, CYP2C9*1, *SLC22A1* (rs594709), and *SLC22A3* (rs2076828). This study moves the question from North America to the most relevant populations in South America, the Andean populations studied in Ecuador. Diabetes is associated in part with poverty-related health problems, which is sometimes related to autochthonous American populations, contributing to the maintenance of the poverty–disease circuit. The main aim is therefore whether there is any peculiarity in terms of pharmacogenetic variants that would allow the optimization of personalized medicine programs in Latin American autochthonous populations [26,27]. In other words, if the Native American component (NATAM) is related to the presence of certain pharmacogenetic variables in diabetic patients from Ecuador in the South American region of the Andes in order to develop Personalized Medicine programs for T2DM patients.

This present study aims to evaluate the distribution of Native American (NATAM), European (EUR), and African (AFR) ancestry in Ecuadorian T2DM patients and its correlation with 19 variants of the seven genes (*CYP2C8*, *CYP2C9*, *CYP2C19*, *SLC22A1, SLC22A2*, *SLC22A3*, and *ABCB1*) that encode the relevant enzymes and transporters implicated in the pharmacogenetics of antidiabetic drugs.

## 2. Results

### 2.1. Genotype and Allelic Frequencies

This study analyzed 297 patients with T2DM from Ecuador. The diplotype and allele frequencies of the cytochromes *CYP2C8*, *CYP2C9*, and *CYP2C19*, as well as the “activity score”, are summarized in Table S1, as well as the allelic and genotypic frequencies and activity score distribution by *CYP2C8, CYP2C9*, and *CYP2C19* within a sample of Ecuadorian T2DM patients (n = 297). The allelic and genotypic frequencies of the SNVs in the *SLC22A1, SLC22A2, SLC22A3*, and *ABCB1* genes are summarized in Table S2.

SNV allelic and genotypic frequencies in *SLC22A1, SLC22A2, SLC22A3*, and *ABCB1* (n = 297) are reported. All analyzed alleles were in Hardy–Weinberg equilibrium, except for SNV rs594709 in the *SLC22A1* gene (*p* = 0.030).

### 2.2. Description of Ancestry in Ecuadorian Patients with T2DM

**Figure 1:**
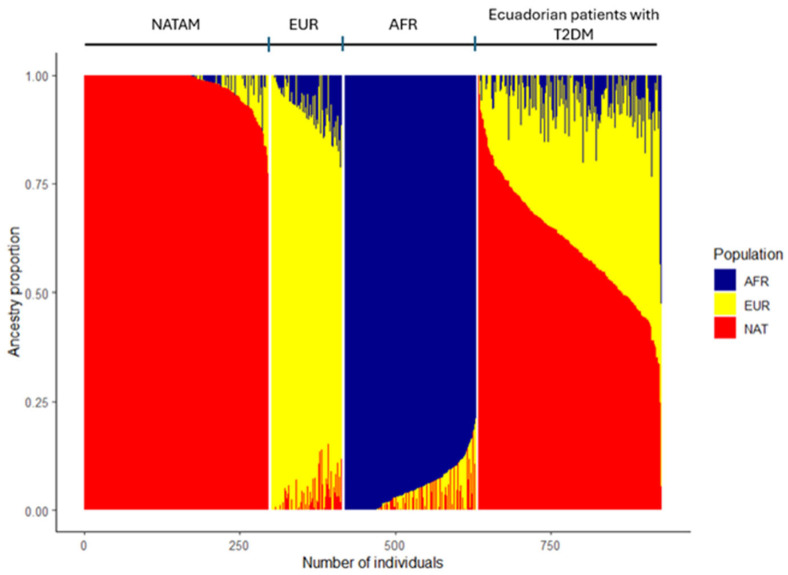
Individual ancestry proportions (k = 3) of Native Americans (n = 296), Spaniards (n = 114) from previous studies [27], Yoruba from 1000 Genomes (AFR, n = 209) [30], and Ecuadorian T2DM patients (n = 294).

The proportion of NATAM, EUR, and AFR ancestry was determined in 294 Ecuadorian patients. The clusters from which ancestry proportions were estimated are shown in Figure 1. In the analysis of the median and the 25th and 75th percentiles of ancestry proportions in our sample, we found that the highest proportion corresponded to NATAM ancestry [61.33% (51.59–70.61)], followed by EUR ancestry [34.48% (25.55–42.36)] and AFR ancestry [2.60% (0.00–7.45)].

### 2.3. Ancestry Inference Among CYP2C8, CYP2C9, and CYP2C19 Diplotypes and Activity Scores

The proportion of NATAM, EUR, and AFR ancestries by groups of individuals with different diplotypes and activity scores of the cytochromes *CYP2C8, CYP2C9*, and *CYP2C19* is summarized in Table 1.

#### 2.3.1. *CYP2C8*

**Figure 2:**
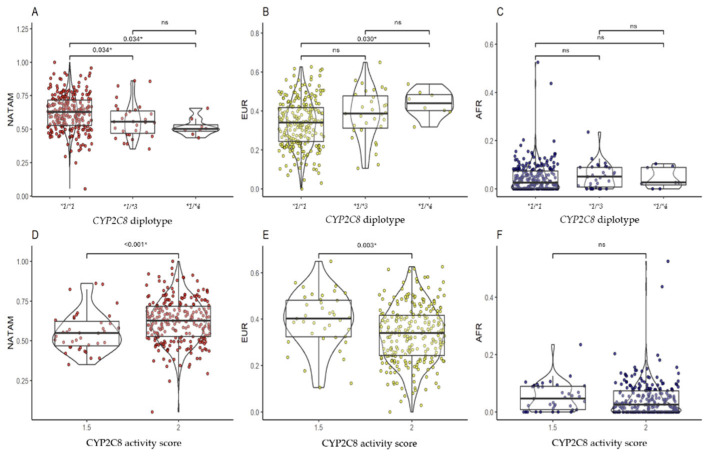
Comparison of ancestry proportions (NATAM, EUR, and AFR) among Ecuadorian T2DM patients grouped by diplotype and activity score of *CYP2C8*. Panels (**A**–**C**) show grouping by *CYP2C8* diplotypes (*1/*1, *1/*3, *1/*4). Panels (**D**–**F**) show grouping by *CYP2C8* activity score (1.5 and 2). The *p*-value corresponds to the post hoc test with Bonferroni adjustment. * Statistical significance (*p* < 0.05); ns: not significant.

Comparing the ancestry proportions among the different *CYP2C8* diplotypes, we found statistically significant differences for NATAM ancestry (*p* = 0.002) and EUR ancestry (*p* = 0.005). Post hoc analysis of *CYP2C8* diplotypes revealed that, for NATAM ancestry, the *1/*1 diplotype was significantly different*1/*3 (pBonferroni = 0.034) and *1/*4 (pBonferroni = 0.034). According to these results, patients with the *1/*1 diplotype had a higher proportion of NATAM ancestry compared to those with the *1/*3 and *1/*4 diplotypes (Figure 2A). In the analysis by *CYP2C8* activity score groups (Figure 2D), patients categorized with an activity score of 2 had a higher proportion of NATAM ancestry compared to those with a score of 1.5 (*p* < 0.001). Similarly, patients carrying the CYP2C8 *1/*4 diplotype exhibited a higher proportion of EUR ancestry compared to those with the *1/*1 diplotype (pBonferroni = 0.030; Figure 2B). In the activity score analysis (Figure 2E), patients with an activity score of 2 showed a lower proportion of EUR ancestry compared to those with a score of 1.5 (*p* = 0.003). No differences were found in AFR ancestry proportions among *CYP2C8* diplotypes (Figure 2C) or activity scores (Figure 2F).

#### 2.3.2. *CYP2C9*

**Figure 3:**
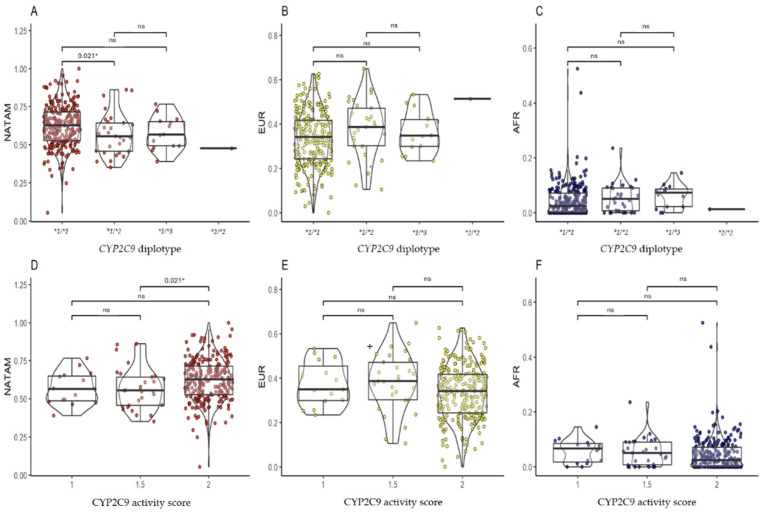
Comparison of ancestry proportion (NATAM, EUR, and AFR) among Ecuadorian T2DM patients grouped by diplotype and activity score of *CYP2C9*. Panels (**A**–**C**) show grouping by *CYP2C9* diplotypes (*1/*1, *1/*2, *1/*3, *2/*2). Panels (**D**–**F**) show grouping by *CYP2C9* activity score (1, 1.5, and 2). The *p*-value corresponds to the post hoc test with Bonferroni adjustment. * Statistical significance (*p* < 0.05); ns: not significant.

In the ancestry inference analysis among *CYP2C9* diplotypes and activity scores, differences were found only for NATAM ancestry (Table 1). In the post hoc analysis, patients carrying the CYP2C9 *1/*1 diplotype showed a higher proportion of NATAM ancestry compared to those with the CYP2C9 *1/*2 diplotype (pBonferroni = 0.021) (Figure 3A). Similarly, patients categorized with an activity score of 2 had a higher proportion of NATAM ancestry compared to those with an activity score of 1.5 (Figure 3D).

#### 2.3.3. CYP2C19

**Figure 4:**
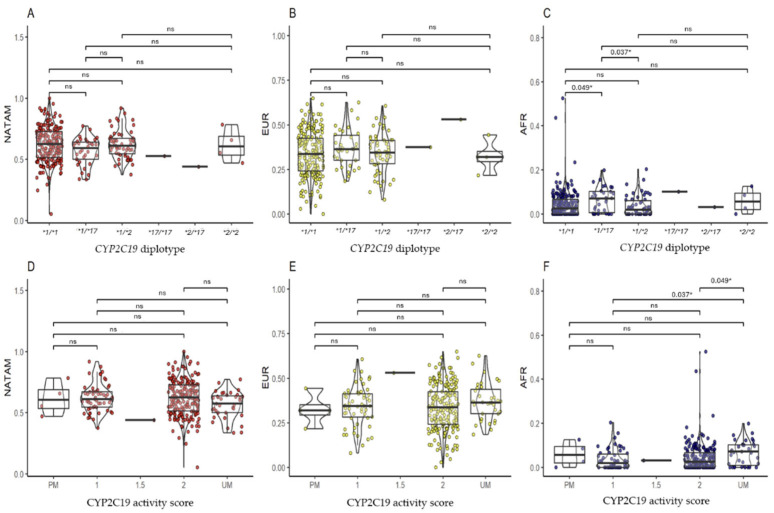
Comparison of ancestry proportion (NATAM, EUR, and AFR) among Ecuadorian T2DM patients grouped by diplotype and activity score of *CYP2C19*. Panels (**A**–**C**) show grouping by *CYP2C19* diplotypes (*1/*1, *1/*17, *1/*2, *17/*17, *2/*17, and *2/*2). Panels (**D**–**F**) show grouping by *CYP2C19* activity score (PM, 1, 1.5, 2, and UM). The *p*-value corresponds to the post hoc test with Bonferroni adjustment. * Statistical significance (*p* < 0.05); ns: not significant.

In the ancestry inference analysis among *CYP2C19* diplotypes and activity score, statistical significance was found only for AFR ancestry. In the post hoc analysis of *CYP2C19* diplotypes (Figure 4C), patients carrying the CYP2C19 *1/*17 diplotype had a higher proportion of AFR ancestry compared to those with CYP2C19 *1/*1 (pBonferroni = 0.049) and CYP2C19 *1/*2 (pBonferroni = 0.037). Despite classifying a *CYP2C19* phenotype based on the alleles carried by the patients, we decided to establish an activity score similar to the current *CYP2D6* phenotype classification, as has been proposed in previous studies [31]. Moreover, in the activity score analysis (Figure 4F), patients categorized as UM (ultra-rapid metabolizers) had a higher proportion of AFR ancestry compared to those with activity scores of 2 (pBonferroni = 0.049) and 1 (pBonferroni = 0.037).

### 2.4. Ancestry Inference Among Transporter SNVs

The inference of NATAM, EUR, and AFR ancestry proportions across groups of individuals with different genotypes of the transporters OCT1, OCT2, OCT3, and *p*-gp, encoded by the *SLC22A1*, *SLC22A2, SLC22A3*, and *ABCB1* genes, respectively, is summarized in Table 2. Statistical significance was found only in the distribution of NATAM, EUR, and/or AFR ancestry for variants in *SLC22A1* (rs72552763, rs594709, rs628031) and *SLC22A3* (rs2076828).

#### 2.4.1. *SLC22A1*

**Figure 5:**
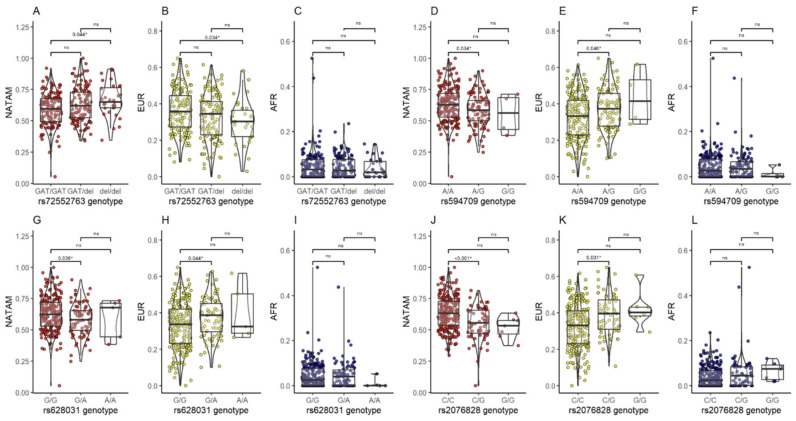
Comparison of ancestry proportion (NATAM, EUR, and AFR) among Ecuadorian T2DM patients grouped by genotypes of *SLC* variants. Panels (**A**–**C**): Grouping by genotypes of rs72552763 in *SLC22A1*. Panels (**D**–**F**): Grouping by genotypes of rs594709 in *SLC22A1*. Panels (**G**–**I**): Grouping by genotypes of rs628031 in *SLC22A1*. Panels (**J**–**L**): Grouping by genotypes of rs2076828 in *SLC22A3*. * Statistical significance (*p* < 0.05); ns: not significant.

For the *SLC22A1* rs72552763 variant, differences in NATAM (*p* = 0.022) and EUR (*p* = 0.017) ancestry proportions were found. Post hoc analysis showed that patients with the del/del genotype had a higher proportion of NATAM ancestry compared to those with the GAT/GAT genotype (pBonferroni = 0.044) (Figure 5A). Regarding EUR ancestry, patients with the GAT/GAT genotype had a higher EUR ancestry proportion than those with del/del (pBonferroni = 0.034) (Figure 5B). For *SLC22A1* rs594709, ancestry differences were found in NATAM (*p* = 0.040) and EUR (*p* = 0.013). Post hoc analysis revealed that patients with the AA genotype had higher NATAM ancestry than those with AG (pBonferroni = 0.034) (Figure 5D). In contrast, EUR ancestry was higher in patients with the AG genotype compared to those with AA (pBonferroni = 0.046) (Figure 5E).

For rs628031, differences were found in NATAM (*p* = 0.042) and EUR (*p* = 0.040) ancestry proportions. Post hoc analysis of NATAM ancestry (Figure 5G) showed that patients with the GG genotype had a higher proportion of NATAM ancestry compared to GA carriers (pBonferroni = 0.036). Conversely, patients with the GA genotype had higher EUR ancestry compared to GG carriers (pBonferroni = 0.044) (Figure 5H).

#### 2.4.2. *SLC22A3*

Statistically significant differences in NATAM (*p* < 0.001) and EUR (*p* < 0.001) ancestry proportions were found for individuals with different genotypes of *SLC22A3* rs2076828. Post hoc analysis revealed that patients with the CC genotype had higher NATAM ancestry (Figure 5J) than those with the CG genotype (pBonferroni < 0.001). Conversely, CG genotype carriers had higher EUR ancestry (Figure 5K) compared to CC carriers (pBonferroni = 0.001).

### 2.5. Correlation Analysis Between Genetic Variants and Ancestry Proportion

To explore correlations between alleles and NATAM, EUR, and AFR ancestry proportions, we conducted Spearman correlation analyses for each cytochrome and transporter allele found to be statistically significant in inferential analyses. Allelic correlation and ancestry proportion analyses were performed for *CYP2C8, CYP2C9, CYP2C19*, and variants of *SLC22A1* (rs72552763, rs594709, rs628031) and *SLC22A3* (rs2076828).

#### 2.5.1. Correlation Analysis for CYP2C8 Variants

The findings of the correlation analysis between alleles (*wt*, *3, *4) and CYP2C8 activity score with NATAM, EUR, and AFR ancestry are summarized in Table S3. Statistically significant correlations were observed between certain *CYP2C8* alleles and NATAM (*wt*, *3, *4) or EUR (*wt* and *4) ancestry, but not AFR.

A positive correlation was found between the CYP2C8 *wt* allele and NATAM ancestry (Rho = 0.193, *p* < 0.001), and negative correlations were observed for CYP2C8*3 (Rho = −0.140, *p* = 0.015) and *4 (Rho = −0.137, *p* = 0.018). EUR ancestry showed a negative correlation with the *wt* allele (Rho = −0.170, *p* = 0.003) and a positive correlation with CYP2C8*4 (Rho = 0.142, *p* = 0.014).

#### 2.5.2. Correlation Analysis for CYP2C9 Variants

The correlation analysis between alleles (wt, *2, *3) and CYP2C9 activity score with NATAM, EUR, and AFR ancestry is summarized in Table S4. NATAM ancestry showed a positive correlation with the *wt* allele (Rho = 0.163, *p* = 0.005) and a negative correlation with the CYP2C9*2 allele (Rho = −0.141, *p* = 0.015). EUR ancestry showed a negative correlation with the *wt* allele (Rho = −0.127, *p* = 0.028) and a positive correlation with CYP2C9*2 (Rho = 0.121, *p* = 0.038). AFR ancestry showed a negative correlation with the *wt* allele (Rho = −0.130, *p* = 0.025).

#### 2.5.3. Correlation Analysis for CYP2C19 Variants

The correlation analysis between alleles (wt, *2, *4, *17) and CYP2C1*9* activity score is summarized in Table S5. Correlation between ancestry proportion and allelic frequency in *CYP2C19* variants. Statistically significant findings were observed only for the CYP2C19*17 allele, which was negatively correlated with NATAM ancestry (Rho = −0.146, *p* = 0.012) and positively correlated with AFR ancestry (Rho = 0.174, *p* = 0.002).

**Figure 6:**
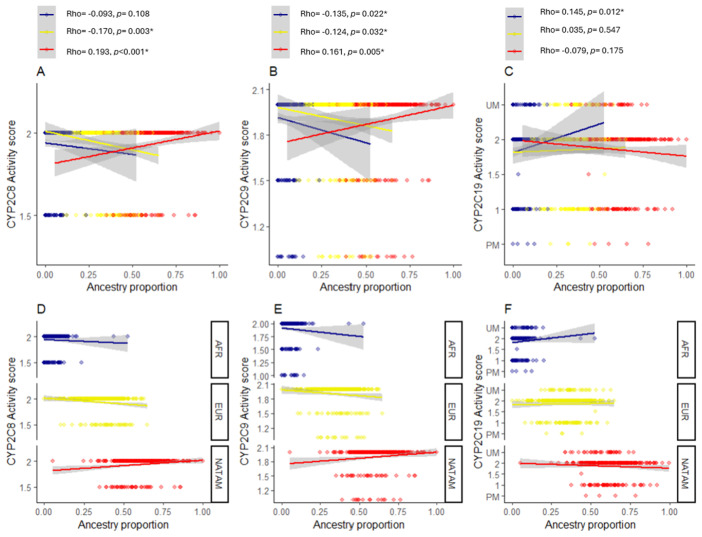
Scatter plot of the activity score of cytochromes encoded by *CYP2C8*, *CYP2C9*, and *CYP2C19*, according to ancestry proportion (NATAM, EUR, and AFR). Lines represent the fitted line from a linear regression model, and grey areas represent the 95% confidence interval. The statistics shown correspond to Spearman’s ordinal correlation for each ancestry proportion. Panels (**A**–**C**): Plots corresponding to *CYP2C8*, *CYP2C9*, and *CYP2C19*, respectively. Panels (**D**–**F**) correspond to the faceted scatter plots according to ancestry proportion in *CYP2C8*, *CYP2C9*, and *CYP2C19,* respectively. Red points correspond to NATAM ancestry, yellow points correspond to EUR ancestry, and blue points correspond to AFR ancestry. * Statistical significance (*p* < 0.05).

To analyze the correlation between enzymatic activity of CYP2C8, CYP2C9, and CYP2C19, a scatter plot was generated for activity scores and ancestry proportions (NATAM, EUR, AFR), adjusted with a linear regression line (Figure 6). For CYP2C8 activity scores (1.5 and 2), a positive correlation with NATAM ancestry (Rho = 0.193, *p* < 0.001) and a negative correlation with EUR ancestry (Rho = −0.170, *p* = 0.003) were found (Figure 6A). For CYP2C9 enzymatic activity (Figure 6B), a positive correlation was found with NATAM ancestry (Rho = 0.161, *p* = 0.005), and negative correlations with EUR ancestry (Rho = −0.124, *p* = 0.032) and AFR ancestry (Rho = −0.135, *p* = 0.022). For CYP2C19, a positive correlation was observed between activity score and AFR ancestry (Rho = 0.145, *p* = 0.012).

#### 2.5.4. Correlation Analysis for Transporters SNVs

The correlation analysis between ancestry proportions (NATAM, EUR, AFR) and SNV alleles (rs72552763, rs594709, rs628031, rs2076828) is summarized in Table S6. For NATAM ancestry, positive correlations were found with the A allele of rs594709 (Rho = 0.147, *p* = 0.011), the G allele of rs628031 (Rho = 0.147, *p* = 0.011), and the C allele of rs2076828 (Rho = 0.261, *p* < 0.001), while a negative correlation was observed with the GAT allele of rs72552763 (Rho = −0.157, *p* = 0.006). For EUR ancestry, a positive correlation was found with the GAT allele of rs72552763 (Rho = 0.162, *p* = 0.005), and negative correlations with the A allele of rs594709 (Rho = −0.169, *p* = 0.003), the G allele of rs628031 (Rho = −0.148, *p* = 0.010), and the C allele of rs2076828 (Rho = −0.230, *p* < 0.001). Only the C allele of rs2076828 was negatively correlated with AFR ancestry (Rho = −0.127, *p* = 0.029).

## 3. Discussion

### 3.1. Ancestry of the Ecuadorian Population

Similar to other Latin American populations, the Ecuadorian population is defined as multiethnic and multicultural, with a complex demographic history of admixture involving Europeans, Native Americans [32], and individuals of African descent since the Spanish colonization. This admixture has shaped the current patterns of ethnic diversity in the population [33].

Within this historical context, our results indicate that the studied population has an ancestry composition of 61.33% NATAM, 34.48% EUR, and 2.60% AFR. These findings are consistent with recent studies reporting a predominance of NATAM ancestry in the Ecuadorian population [33,34].

When compared to other Latin American populations, a high proportion of NATAM ancestry has also been observed in Mexico [28] and Peru [35]. In contrast, populations from Colombia [36] and Brazil [37] exhibit a predominance of EUR ancestry. Caribbean populations, such as those in the Dominican Republic and Puerto Rico, on the other hand, present a significant proportion of AFR ancestry [38,39]. This genetic diversity is associated with interindividual variability in response to pharmacological therapies [40]. This constitutes a current challenge in pharmacology, as therapeutic response to various treatments has been shown to vary substantially across global populations [41]. One of the contributing factors is the frequency distribution of pharmacogenetic variants among different ethnic groups [33].

### 3.2. Ancestry and Pharmacogenetics of CYP450

#### 3.2.1. *CYP2C8*

In the analysis of molecular ancestry and genetic variants of *CYP2C8*, significant differences were found in relation to the NATAM and EUR ancestral components. Specifically, carriers of the *1 allele exhibited predominantly NATAM ancestry, which was less prevalent among carriers of the *3 and *4 alleles. These results suggest a positive association between the CYP2C8*1 variant and NATAM ancestry, while the CYP2C8*4 variant may correlate with EUR ancestry. None of the alleles showed a significant association with AFR ancestry.

The allele frequencies of CYP2C8*3 and CYP2C8*4 in the studied population were 5.21% and 1.51%, respectively. These values are slightly lower than previously reported frequencies, where CYP2C8*3 was found at 8.1% in the Ecuadorian population [42]. In contrast, public databases report a somewhat higher frequency of this variant in European populations (~10%) [43].

*CYP2C8* variability may significantly impact the pharmacokinetics, therapeutic response, and toxicity of a wide range of drugs, including antidiabetics [44]. Notably, the American Diabetes Association (ADA) and the European Association for the Study of Diabetes (EASD) recommend the use of TZDs, particularly pioglitazone and rosiglitazone, for the treatment of diabetes [16]. These drugs are primarily metabolized by *CYP2C8* [16]. The CYP2C8*4 variant is considered a loss-of-function allele; however, the enzymatic activity of CYP2C8*3 remains unclear, as it has been shown to be substrate-specific. Some studies have reported no reduction in pioglitazone metabolism, whereas others have demonstrated increased metabolism of TZDs and a 36% reduction in rosiglitazone plasma concentration among healthy carriers of CYP2C8*3. This reduction may lead to diminished therapeutic efficacy, with higher levels of glycated hemoglobin observed in CYP2C8*3 carriers compared to *1/*1 individuals [16]. Additionally, adverse effects such as edema and weight gain have been reported more frequently in T2DM patients carrying the *3 allele compared to wild-type individuals [19].

Currently, no specific pharmacogenetic clinical guidelines exist for *CYP2C8*. However, this enzyme is included alongside *CYP2C9* in the pharmacogenetic guideline for non-steroidal anti-inflammatory drugs (NSAIDs) [45] because the CYP2C8*3 allele is in strong linkage disequilibrium with the CYP2C9*2 allele [46,47]. Consequently, for *CYP2C8* activity classification, we adopted the same system used for *CYP2C9* [45], where CYP2C8*3 and CYP2C8*4 are assigned an activity score of 1.5. More recently, an alternative phenotypic classification for *CYP2C8* has been proposed, defining UMs as *3/*3 individuals, rapid metabolizers (RMs) as *1/*3, normal metabolizers (NMs) as *1/*1, intermediate metabolizers (IMs) as *1/*4, and poor metabolizers (PMs) as *4/*4 [48]. This discrepancy arises from the previously mentioned substrate-specific activity of *CYP2C8*, leading to ongoing debate regarding the functional classification of CYP2C8*3. In fact, one study reported that depending on whether CYP2C8*3 is considered a normal- or decreased-function allele, the proportion of NMs in Ecuador could range from 100% to approximately 90%, with the remaining 10% being classified as IMs [49].

Given the absence of clinical guidelines for *CYP2C8* genotyping in diabetic patients, we propose incorporating ancestry as a key factor to enhance therapeutic precision and reduce adverse reactions to hypoglycemic agents—one of the most significant concerns for current diabetic populations [10]. Therefore, due to the high proportion of NATAM ancestry in the Ecuadorian population and its association with the CYP2C8*1 variant (wild type), the present results suggest that TZDs may represent a safe and effective treatment option for Ecuadorian patients with T2DM. This supports the consideration of ethnogeographic background in *CYP2C8* variability profiling.

#### 3.2.2. *CYP2C9*

In the analysis of the relationship between molecular ancestry and *CYP2C9* genetic variants, significant differences were observed exclusively with the NATAM ancestral component. A correlation was found between *1/*1 individuals and higher NATAM ancestry compared to *1/*2 individuals. Consequently, individuals with a genotype-inferred phenotype classified as NMs exhibited a greater proportion of NATAM ancestry than those classified as IMs. These findings support previous studies demonstrating that the CYP2C9*2 variant is more prevalent among individuals with EUR ancestry, while the CYP2C9*3 variant is commonly found in South Asian populations [50].

In the Ecuadorian population, the allele frequencies of CYP2C9*2 and CYP2C9*3 were 5.23% and 2.36%, respectively, resulting in an estimated prevalence of approximately 15% for intermediate metabolizers. These relatively low frequencies are consistent with earlier findings, where the *2 and *3 allele frequencies were reported as 2.44% and 2.83%, respectively [42,51], with a comparable IM prevalence of about 14% [51]. However, our results indicate a slightly higher frequency than that reported in a more recent study [50]. As in previous research, no PMs were identified in the population [51].

Similar frequencies of CYP2C9*2 and CYP2C9*3 have been reported in other Latin American populations, including 4.6% and 6.2% in Peru, and 3.83% and 2.82% in a Mexican T2DM population [28]. However, the highest reported frequencies of these variants in South America are found in Uruguay, Colombia, and Brazil [50], which is consistent with the greater EUR ancestry observed in those populations.

According to the Clinical Pharmacogenetics Implementation Consortium (CPIC) guidelines, the enzymatic activity of *CYP2C9*, and therefore the classification of individuals as NMs (*1/*1), IMs (*1/*2, *1/*3, *2/*2), or PMs (*2/*3, *3/*3), is determined by the presence of the *1, *2, and *3 alleles. These classifications are particularly relevant for the prescription of drugs such as NSAIDs [45], phenytoin [52], and warfarin [53]. Previous studies have reported adverse reactions to SUs in carriers of *CYP2C9* decreased-function alleles, who are more likely to experience secondary hypoglycemia [18]. This finding is supported by a recent meta-analysis showing that T2DM patients carrying CYP2C9*2 had a higher risk of hypoglycemic episodes due to reduced metabolic activity, ultimately compromising SU efficacy [54].

Nonetheless, no consensus has been reached regarding the impact of *CYP2C9* genotype on SU efficacy. Some studies report better therapeutic outcomes in *1/*1 patients compared to *1/*3 and *3/*3 carriers [55], which would favor the Ecuadorian population due to its high frequency of CYP2C9*1. Other studies suggest that CYP2C9*3 carriers may achieve better glycemic control, possibly due to prolonged drug half-life [56,57]. This inconsistency in findings may stem from several factors, including study design heterogeneity, differences in hypoglycemia definitions, patient age, the specific SU assessed, or sample size limitations [58]. Despite these discrepancies, SU-induced hypoglycemia remains a current challenge in T2DM management, as it can lead to adverse effects ranging from mild discomfort or treatment withdrawal to serious morbidity [59].

Therefore, genotyping *CYP2C9* variants specific to each population may be crucial, particularly during the initiation of SU therapy, in order to optimize treatment efficacy and reduce the risk of ADRs.

#### 3.2.3. *CYP2C19*

In the inference analysis between the *CYP2C19* gene and molecular ancestry, significant differences were observed only with the AFR ancestral component. Additionally, in the diplotype analysis, individuals carrying the CYP2C19 *1/*17 genotype exhibited a higher proportion of AFR ancestry compared to CYP2C19 *1/*1 and CYP2C19 *1/*2 individuals. These findings are consistent with previous studies reporting that the increased function CYP2C19*17 allele and this UM phenotype are negatively associated with NATAM ancestry, helping to explain the variability in allele frequencies across Latin America [27], and positively associated with EUR and AFR ancestry [38].

In the studied population, the frequencies of CYP2C19*2 and CYP2C19*17 were 11.14% and 6.25%, respectively. These frequencies support previous findings reporting a loss-of-function (*2) allele frequency of approximately 12% in Ecuador, and an increased-function (*17) allele frequency of 2.10% [60]. Other studies have reported slightly higher CYP2C19*17 frequencies (9.5%) in Ecuador [61], although these are still lower than those reported in European or African populations [62].

Based on *CYP2C19* genotypes, the extrapolated phenotypic distribution was 1.35% PMs, 11.82% IMs, and approximately 20% UMs. Interestingly, the observed frequency of UMs is notably lower than previously reported values (41.4%), while the PM frequency was higher (0.7%) [42]. Although SUs are primarily metabolized by *CYP2C9*, *CYP2C19* also contributes to their metabolism [15]. A study conducted in China proposed that *CYP2C19* may play an even more significant role than *CYP2C9* in the metabolism of gliclazide [63], suggesting increased efficacy and reduced ADRs. However, that study did not report the presence of CYP2C19*17 carriers [64].

According to CPIC guidelines for *CYP2C19* metabolized drugs, such as clopidogrel [65], tricyclic antidepressants [66], and selective serotonin reuptake inhibitors (SSRIs) [67], the *1 allele is classified as normal function, *17 as increased function, and *2 and *4 as decreased function alleles. These guidelines provide drug dosing recommendations and strategies to minimize ADRs based on genotype-inferred phenotypes.

Despite the absence of pharmacogenetic guidelines for antidiabetic drugs, SU remains part of combination therapy with metformin when glycemic control is not adequately achieved [6]. Since these drugs are metabolized by highly polymorphic enzymes such as *CYP2C9* and *CYP2C19*, variability in enzyme function can lead to reduced drug efficacy and the occurrence of ADRs, including severe secondary hypoglycemia [18].

### 3.3. Ancestry and OCTs

Our results from the analysis of ancestry distribution in variants of the OCTs show that the ancestral component is unevenly distributed among the genotypes of variants rs72552763, rs594709, and rs628031 of the *SLC22A1* gene and the rs2076828 variant of the *SLC22A3* gene. These findings contrast with previously obtained results by RIBEF collaboration in a study conducted in Mexican patients diagnosed with T2DM [28], where differences were only found in the distribution of the ancestry proportion among the genotypes of variants rs2076828 and rs628031.

#### 3.3.1. *SLC22A1*

In the allelic correlation and ancestry proportion analysis, we found that the GAT allele of the rs72552763 variant is negatively correlated with NATAM ancestry and positively correlated with EUR ancestry. These findings are consistent with databases such as the 1000 Genomes Project, where the highest frequency of the deletion is found in populations from the Americas, specifically Mexicans from Los Angeles, California, and Peruvians from Lima [68], with results very similar to those in our sample and those reported in Mexican mestizos diagnosed with T2DM [28]. These findings suggest that populations in the Americas may have a higher prevalence of the variant allele of rs72552763, which in turn suggests possible pharmacogenetic implications. Some studies have reported implications in the pharmacokinetics of metformin associated with the presence of the del allele of rs72552763, among which are a decrease in the hepatic volume of distribution of metformin in del/del genotype carriers compared with carriers of the GAT allele [69], a decrease in metformin steady-state in T2DM patients carrying the variant, including rs72552763 [70], as well as pharmacokinetic alterations of other cationic drugs, such as decreased transport of ranitidine [71] and reduced clearance of morphine [72] observed in del/del genotype carriers. This suggests a possible decrease in OCT-1 transporter function in the presence of the del allele of rs72552763, which may have clinical implications, particularly in terms of therapeutic efficacy and the occurrence of adverse reactions associated with metformin or cationic drugs. Some studies have reported higher HbA1c levels in Mexican T2DM patients treated with metformin who carry the *del* allele [29]. Furthermore, in a longitudinal analysis conducted in Mexican T2DM patients treated with metformin monotherapy, the del allele has also been associated with a shorter time to HbA1c non-control compared with patients carrying the GAT/GAT genotype [73]. Similarly, it has been reported that patients carrying the GAT/del genotype have a higher risk of metformin-associated adverse reactions in Egyptian patients diagnosed with T2DM [74], where the frequency of the del allele is particularly low [68].

Our findings on rs594709 suggest a positive correlation between the presence of the A allele and NATAM ancestry and a negative correlation of the A allele with EUR ancestry. These findings are consistent with what has been reported in populations from the Americas, where the A allele is the most frequent (78%), especially in Mexicans and Peruvians, where this figure reaches 86.7% and 90.0%, respectively [68].

Studies conducted in diabetic patients considering the possible clinical implications of rs594709 are limited, and no association has been reported with the time to HbA1c non-control in Mexican T2DM patients [73]. However, in a study where an adjustment was made for another variant such as rs2289669, specifically with the G allele, it was found that the presence of the AA genotype of rs594709 and the G allele of rs2289669 may be associated with a decrease in fasting glucose, without having a significant impact on HbA1c [75]. Another study conducted in Mexican T2DM patients followed for 12 months found no differences in HbA1c levels among rs594709 genotypes; however, after adjusting for sex, time since diabetes diagnosis, and abdominal circumference, it was found that patients carrying the GG genotype showed higher HbA1c levels compared with carriers of the A allele [76]. Based on the above, although we found a significant correlation between NATAM, EUR ancestry, and the A allele of rs594709, the clinical implication of this variant in the clinical pharmacogenetics of diabetes may be modest.

The findings in this work regarding rs628031 suggest that the G allele is positively correlated with NATAM ancestry and negatively with EUR ancestry, consistent with what has been reported in the 1000 Genomes Project, as the population in the Americas has the highest prevalence of the G allele, reaching 88.3% and 90.0% in Mexicans and Peruvians, respectively [68]. Additionally, these frequencies are similar to those found in our study as well as in other studies conducted in the Mexican population [28].

The effect of the rs628031 variant on h-OCT1 transporter function has been studied, finding that the loss of transporter function with the presence of the rs628031 (M420V) variant is approximately 10% [77], conditioning a possible increase in metformin concentration, therapeutic efficacy, and adverse reactions in carriers of the A variant. However, no impact has been found on metformin C_ss (metformin steady-state concentration)_ among the different genotypes of rs628031 [78]. The clinical impact of this variant has been studied in Han Chinese patients treated with metformin, where AA carriers showed a greater reduction in HbA1c [79]. However, in a study conducted in diabetic patients from northern Mexico, it was found using an over-dominant model that carriers of GG+AA genotypes of rs628031 have higher HbA1c levels compared with GA carriers [80].

#### 3.3.2. *SLC22A2*

In this study, we analyzed the rs316019 variant of *SLC22A2*; however, no significant correlation was found between rs316019 alleles and the proportion of NATAM, EUR, or AFR ancestry. According to data from the 1000 Genomes Project, the minor allele frequency in the American population, specifically in Mexico, is 5.5% and in Peru 5.3%, values comparable to that observed in the present work (3.5%) for the A allele of rs316019. In contrast, in Europe this frequency ranges between 6.1% and 13.1% [68]. Although OCT2 is responsible for most of the renal clearance of metformin [81], some studies have not found an association with the drug’s therapeutic response, or suggest that the effect may be small [18]. Another study reported that the AUC_0–48h_ of metformin tends to be higher in individuals carrying the wild-type genotype compared to rs316019 heterozygotes; however, these differences were not statistically significant [82]. In contrast, other studies have identified an association between metformin therapeutic response and codominant models involving rs316019 of *SLC22A2* and rs12943590 of *SLC47A2*, as these variants appear to influence HbA1c levels [83]. Nevertheless, the low prevalence of rs316019 and the modest effect reported in metformin users suggest that its relevance at the population level is limited.

#### 3.3.3. *SLC22A3*

In this work, we found a positive correlation between the C allele of rs2076828 and NATAM ancestry, while the correlation was negative with EUR and AFR ancestry. This finding is consistent with what has been reported in the 1000 Genomes Project database, as the population with the highest frequency of the C allele belongs to the American continent [68].

Through an in vitro assay that used luciferase activity as a biomarker of gene expression, it was determined that the presence of the G allele of rs2076828 was associated with significantly lower luciferase activity compared with the reference C allele. In addition, it was observed that the minor G allele is associated with a reduced response to metformin in healthy subjects; however, the presence of the rs2076828 variant does not seem to have a significant effect on the pharmacokinetic parameters of metformin [84]. This may be because the OCT3 transporter is predominantly expressed in skeletal muscle, where, through increased AMP-Activated Protein Kinase (AMPK) activity in muscle, metformin improves glucose uptake and increases muscle glycogen [85]. In turn, OCT3 expression is moderate in the basolateral membrane of the proximal convoluted tubule, participating in metformin elimination; however, its role is less important than that of OCT2, which plays a key role in the excretion of pharmacological and endogenous substrates [86]. In this sense, it is possible that in populations with a high prevalence of the G allele, the therapeutic response to metformin may be reduced.

#### 3.3.4. *ABCB1*

In this study, we analyzed three *ABCB1* polymorphisms (rs1045642, rs2032582, and rs1128503) but found no evidence of correlation between the alleles or genotype distributions of these variants and the proportion of NATAM, EUR, or AFR ancestry. *ABCB1* encodes P-gp, a transporter protein that facilitates the efflux of metformin [87]. However, to the best of our knowledge, the influence of these variants on the clinical efficacy of metformin remains limited. The absence of statistically significant differences in ancestry proportions, combined with the lack of correlation between these genotypes and ancestry, reinforces the notion that not all genetic variants with potential pharmacogenetic relevance necessarily have population-level or structural significance.

The characterization of genetic variants in Latin American populations associated with drug response is essential for identifying groups at risk of potential toxicity when administered specific medications. This knowledge is particularly valuable when translating clinical data from foreign populations to local contexts [88], as previous studies have reported associations between T2DM and non-European ancestry in Latino populations [89,90]. In fact, determining the frequencies of these molecular variants in Latin American populations, particularly in healthy individuals, will allow for the assessment of the safety and efficacy of antidiabetic drugs in these populations.

## 4. Materials and Methods

### 4.1. Study Design

This was an observational study conducted in accordance with the Declaration of Helsinki, and it was approved by the Bioethics Committee at the Universidad San Francisco de Quito (2017–077T–06/27/2017). All participants gave written informed consent.

### 4.2. Inclusion and Exclusion Criteria

The patients recruited for this study were diagnosed with T2DM and undergoing chronic treatment with SU or metformin. Patients of both genders aged between 35 and 65 years were included. Exclusion criteria were as follows: patients diagnosed with type 1 diabetes, as well as those with a recognized genetic syndrome of insulin resistance; chronic gastrointestinal diseases associated with malabsorption such as chronic pancreatitis, alcoholism, or drug abuse; pregnant women; kidney, liver, or thyroid disease, and concomitant treatment with corticosteroids or estrogens.

### 4.3. Data Collection

This study analyzed 297 patients with T2DM attending to the outpatient’s clinic at the “Dispensario Central”, which belongs to the Social Security Institute in Quito, Ecuador between September 2017 and August 2018.

### 4.4. Genotyping Procedure

The DNA was isolated and purified from blood samples using a QIAmp DNA extraction kit (Qiagen, Hilden, Germany). Genotyping for the different CYP450 and transport variants (Table S7: Analysis allelic variants with their respective PCR-TR probes across Ecuadorian T2DM patients (n = 297) was performed using commercially available genomic DNA Taqman^®^ assays (Applied Biosystems, Foster City, CA, USA). Genotypes were assigned according to the presence of “key” SNVs associated with the relevant alleles (Table S7). All assays included positive (heterozygous and/or homozygous) and negative (no DNA) control samples from previous studies of our group. Plates were read with an ABI 7300 real-time PCR system (Applied Biosystems, Foster City, CA, USA), and the following thermocycling conditions were applied for all assays: 10 min for initial denaturation at 95 °C, followed by 40 denaturation cycles of 15 s at 92 °C and annealing at 60 °C for 1 min. Allele discrimination lasted 30 s at 60 °C. Genotype-based phenotype predictions were assigned to CYP2C8 and CYP2C9 as follows: individuals carrying either two non-functional alleles or one non-functional allele plus a reduced function allele were PMs, with an assigned activity score of 0–0.5. IMs were those individuals with either two reduced function alleles or a normal function allele plus a reduced function or non-functional allele, with an activity score of 1–1.5. NMs carry two normal function alleles, with an activity score of 2 [91]. There is no current activity score assignment consensus about CYP2C19. Individuals are rather classified according to their *CYP2C19* genotype; however, we employed a classification of the different metabolizer phenotypes: PMs carry two non-functional alleles, activity score 0; IMs carry one normal function allele plus one non-functional allele, or an increased function allele plus one non-functional allele, activity score 1–1.5; NMs carry two normal function alleles, activity score 2; rapid (gRMs) and UMs carry one normal function allele plus an increased function allele or two increased function alleles, respectively. These two latter groups are integrated into a single UM with an activity score of >2 [65]. This phenotype group classification based on genotype and/or activity score has been developed from published CPIC [65,91].

### 4.5. Genomic Ancestry Analysis

Individual genomic ancestry was determined in 297 individuals from Ecuador. Out of these, 3 individuals did not register all 90 ancestry markers; thus, no ancestry proportion was determined thereby. The AFR, EUR, and NATAM components were inferred by genotyping 90 ancestry informative markers (AIMs) from the same panel used in previous studies [28,38] AIMs genotyping was performed at the National Genotyping Centre (CEGEN) at Santiago de Compostela, Spain, using the Sequenom (San Diego, CA, USA) platform. Individuals from the three parental populations were also inserted in the final database: 114 Spaniards and 296 Peruvian Native Americans from RIBEF-CEIBA [27] and 209 African Yoruba individuals from the 1000 Genomes Project [30]. The complete databases were transformed to ped map format using GLU 1.0b2 software (https://code.google.com/archive/p/glu-genetics/; accessed on 25 October 2024) and then analyzed in the Admixture software [92] in an unsupervised mode, assuming a tri-hybrid model (k = 3).

### 4.6. Statistical Analysis

The statistical analysis and figures were generated using R-4.2.0 (available at: https://www.R-project.org/; accessed on 18 February 2025). The analysis was carried out across three distinct phases: (i) descriptive, (ii) inferential, and (iii) correlation.

#### 4.6.1. Descriptive Analysis

A description of allelic and genotypic frequencies was carried out for the variants under investigation. In the case of CYP2C8, CYP2C9, and CYP2C9, activity scores are also reported. The Hardy–Weinberg equilibrium was determined through Pearson’s Chi-squared test (Table S1: Allelic and genotypic frequencies and activity score distribution by CYP2C8, CYP2C9, and CYP2C19 within a sample of Ecuadorian T2DM patients (n = 297) and Table S2: SNV allelic and genotypic frequencies in *SLC22A1*, *SLC22A2*, *SLC22A3*, and *ABCB1* (n = 297)).

#### 4.6.2. Inferential Analysis

Ancestry proportion of NATAM, EUR, and AFR components were described and grouped according to the SNVs analyzed. In the case of *CYP2C8*, *CYP2C9,* and *CYP2C19*, ancestry proportions were grouped according to each activity score. Normality tests were performed through Shapiro–Wilk or Kolmogorov–Smirnov test. Inferential analyses were performed through Kruskal–Wallis test and Mann–Whitney’s U test for the case of 2 independent groups. Adjustment of *p*-values for multiple comparisons was performed employing the Bonferroni correction method and False Discovery Rate through Benjamini–Hochberg test. The significance value was *p* < 0.05.

#### 4.6.3. Correlation Analysis

Across statistically significant transporters and cytochromes (*CYP2C8*, *CYP2C9*, *CYP2C19*, rs72552763, rs594709, rs628031, and rs2076828), Spearman’s rank correlation coefficient was determined by either the genotype’s allele or each individual’s diplotype (0, 0.5, and 1) and the ancestry proportion (NATAM, EUR, or AFR). The significance value was *p* < 0.05. A Spearman’s correlation analysis was performed between ancestry proportion and the activity score of *CYP2C8*, *CYP2C9*, and *CYP2C19*. A scatter plot with a regression line and 95% confidence interval was generated using the function *lm()* in stats package (v4.2.0), through method = (lm) from geom_smooth in ggplot2 (v3.5.1). Spearman’s coefficient correlation was reported, and the significance value was *p* < 0.05.

## 5. Conclusions

Among the studied Ecuadorian population of T2DM patients, the proportion of NATAM ancestry (61.3%) is higher than the EUR (34.5%) or AFR component, which is of very little relevance (2.6%%). Regarding the relevance of this ancestral component of the native population of Ecuador (NATAM), *CYP2C8* and *CYP2C9* (*1/*1 diplotype) and activity score of 2 have been related. No reference has been found for *CYP2C19* (which does appear for the AFR component). In relation to the transport-related NATAM ancestry, it has been related to *SLC22A1* (rs594709 and rs628031) and *SLC22A3* (rs2076828) genes. An association was not found between *SLC22A2* and *ABCB1* with NATAM ancestry.

The results of this study highlight the relevance of considering genomic ancestry in the implementation of clinical pharmacogenetics for T2DM to ensure patients receive safe and effective antidiabetic treatment. By identifying differences among genotypes of the main drug-metabolizing enzymes and transporters involved in antidiabetic drug disposition, and by detecting a correlation between the distribution of these variants and the assignment of the activity score for drugs such as metformin, TZDs, and SUs, this study underscores the importance of a personalized medicine approach to T2DM in Ecuador.

### The Limitations of the Study

In future studies, we aim to analyze the clinical relevance in patients diagnosed with T2DM by collecting clinical, biochemical, and pharmacological parameters, which were not included in the present study. Furthermore, we intend to increase the study sample size by incorporating diverse ethnic groups from across Ecuador, rather than limiting the population to patients from Quito.

## Figures and Tables

**Table 1:** Distribution of ancestry percentage across different genotypes and activity scores of CYP2C8, CYP2C9, and CYP2C19 in Ecuadorian patients diagnosed with T2DM (n = 294).

	NATAM	EUR	AFR		NATAM	EUR	AFR
*CYP2C8*				Activity score			
*1/*1	62.61 (52.71–71.70)	33.88 (24.35–41.58)	2.48 (0.00–7.31)	1	-	-	-
*1/*3	55.41 (46.78–63.68)	38.58 (31.13–47.70)	5.01 (0.77–8.92)	1.5	54.83 (46.85–62.21)	40.23 (32.32–48.16)	4.58 (0.77–8.92)
*1/*4	49.98 (48.43–53.26)	43.80 (40.05–48.37)	2.54 (1.55–8.87)	2	62.61 (52.71–71.70)	33.88 (24.35–41.58)	2.48 (0.00–7.31)
*3/*4	-	-	-				
*p^KW^*	0.002 *	0.005 *	0.262	*p^U^*	<0.001 *	0.003 *	0.109
*CYP2C9*							
*1/*1	62.55 (52.67–71.49)	34.09 (24.34–41.68)	2.40 (0.00–7.06)	1	56.36 (48.72–64.91)	34.89 (29.90–45.35)	6.61 (1.72–8.45)
*1/*2	55.51 (45.76–64.34)	38.58 (30.19–47.06)	5.01 (0.68–9.01)	1.5	55.51 (45.76–64.34)	38.58 (30.19–47.07)	5.01 (0.68–9.01)
*1/*3	56.63 (49.29–65.18)	34.72 (29.79–41.99)	7.18 (2.18–8.59)	2	62.55 (52.66–71.49)	34.09 (24.34–41.68)	2.40 (0.00–7.06)
*2/*2	-	-	-				
*2/*3	-	-	-				
*p^KW^*	0.031 *	0.132	0.053	*p^KW^*	0.021 *	0.097	0.070
*CYP2C19*							
*1/*1	62.42 (51.50–72.79)	33.63 (24.07–42.50)	2.44 (0.00–6.60)	PM	60.54 (53.34–68.82)	31.93 (29.27–35.16)	5.50 (1.90–9.47)
*1/*2	60.94 (54.63–67.25)	34.44 (28.14–41.31)	1.89 (0.00–5.97)	1	60.94 (54.63–67.25)	34.44 (28.14–41.31)	1.89 (0.00–5.97)
*1/*4	-	-	-	1.5	-	-	-
*1/*17	58.93 (50.04–64.08)	36.27 (30.13–44.09)	6.92 (0.58–10.27)	2	62.42 (51.50–72.79)	33.63 (24.07–42.50)	2.44 (0.00–6.60)
*2/*2	60.54 (53.34–68.82)	31.93 (29.27–35.16)	5.50 (1.90–9.47)	UM	57.31 (50.10–63.82)	36.33 (30.15–43.71)	7.06 (0.92–10.19)
*17/*17	-	-	-				
*2/*17	-	-	-				
*p^KW^*	0.183	0.601	0.025 *	*p^KW^*	0.183	0.601	0.025*

*^KW^*, Kruskal–Wallis test; *^U^* Mann–Whitney’s U test; * Statistical significance (*p* < 0.05). Showing median and interquartile ranges (p25–p75). PM: Poor metabolizers; UM: Ultra-rapid metabolizers.

**Table 2:** Ancestry distribution percentage across different genotypes of *SLC22A1*, *SLC22A2*, *SLC22A3*, and *ABCB1* among Ecuadorian T2DM patients (n = 294).

Gene	ID	Genotype	NATAM	EUR	AFR
*SLC22A1*	rs72552763	GAT/GAT	59.61 (49.30–67.77)	35.66 (27.29–44.39)	3.13 (0.00–7.51)
		GAT/del	61.96 (52.55–72.83)	34.42 (22.95–41.17)	2.56 (0.00–7.55)
		del/del	64.98 (60.31–76.51)	30.11 (21.94–36.37)	1.89 (0.00–6.70)
		*p^KW^*	0.022 *	0.017 *	0.842
	rs622342	A/A	60.46 (49.38–67.91)	35.53 (26.25–43.23)	2.70 (0.00–7.86)
		A/C	60.28 (51.47–71.26)	35.30 (24.37–42.77)	2.77 (0.00–7.35)
		C/C	64.98 (60.31–76.51)	30.11 (21.94–36.37)	1.89 (0.00–6.70)
		*P^KW^*	0.247	0.243	0.397
	rs12208357	C/C	61.41 (51.78–70.61)	34.44 (25.55–42.08)	2.62 (0.00–7.39)
		C/T	51.21 (44.62–64.92)	44.73 (34.13–48.11)	0.66 (0.93–8.83)
		T/T	-	-	-
		*p^U^*	0.339	0.265	0.884
	rs2282143	C/C	61.41 (51.78–70.61)	34.44 (25.55–42.08)	2.62 (0.00–7.39)
		C/T	51.21 (44.62–64.92)	44.73 (34.13–48.11)	0.93 (0.66–8.83)
		T/T	-	-	-
		*p^U^*	0.339	0.265	0.884
	rs594709	A/A	62.58 (52.90–71.94)	33.17 (22.91–41.83)	2.57 (0.00–7.76)
		A/G	58.46 (49.93–66.18)	37.13 (27.70–45.61)	3.38 (0.00–6.57)
		G/G	56.09 (42.93–68.57)	41.27 (31.42–53.12)	0.00 (0.00–1.31)
		*p^KW^*	0.040 *	0.013 *	0.298
	rs683369	C/C	61.10 (51.52–70.60)	34.53 (24.27–42.47)	2.60 (0.00–7.68)
		C/G	61.96 (52.16–70.55)	34.13 (27.19–41.71)	3.14 (0.00–6.61)
		G/G	-	-	
		*p^U^*	0.843	0.625	0.849
	rs628031	G/G	62.21 (52.85–72.00)	33.63 (23.17–42.00)	2.47 (0.00–7.64)
		G/A	57.96 (49.51–65.48)	38.47 (29.54–45.10)	4.02 (0.00–6.96)
		A/A	67.71 (44.48–71.17)	32.28 (28.82–50.25)	0.00 (0.00–0.19)
		*p^KW^*	0.042 *	0.040 *	0.143
*SLC22A2*	rs316019	C/C	61.97 (51.93–70.79)	34.33 (25.55–41.90)	2.54 (0.00–7.36)
		C/A	54.99 (46.89–66.91)	39.39 (26.97–44.43)	5.26 (1.03–10.60)
		A/A	-	-	-
		*p^U^*	0.163	0.369	0.120
*SLC22A3*	rs2076828	C/C	63.16 (53.56–72.81)	32.89 (22.99–40.97)	2.31 (0.00–6.61)
		C/G	55.40 (47.32–65.61)	39.52 (31.02–47.14)	4.32 (0.00–8.31)
		G/G	53.25 (46.62–57.56)	40.07 (38.26–43.06)	7.32 (2.70–9.14)
		*p^KW^*	<0.001 *	<0.001 *	0.560
*ABCB1*	rs2032582	G/G	57.27 (49.30–65.98)	36.21 (28.09–44.97)	3.57 (0.00–7.35)
		G/A	60.11 (47.98–67.88)	36.29 (28.13–47.83)	3.84 (1.77–9.51)
		A/A	-	-	-
		G/T	61.33 (50.31–70.44)	34.39 (26.05–42.52)	2.10 (0.00–7.51)
		T/T	62.73 (54.13–72.24)	33.70 (24.05–41.62)	2.22 (0.00–6.44)
		T/A	66.02 (60.04–71.07)	30.90 (23.53–35.07)	3.09 (0.00–7.99)
		*p^KW^*	0.139	0.181	0.623
	rs1128503	C/C	57.93 (49.23–65.52)	36.37 (30.03–44.99)	3.59 (0.00–7.19)
		C/T	60.95 (50.37–70.38)	33.88 (25.74–41.68)	2.31 (0.00–7.75)
		T/T	63.16 (53.14–72.79)	33.18 (23.40–41.76)	2.21 (0.00–6.56)
		*p^KW^*	0.072	0.127	0.481
	rs1045642	C/C	58.04 (49.91–67.67)	34.72 (28.91–44.13)	3.48 (0.00–6.96)
		C/T	61.26 (50.63–69.90)	34.42 (25.96–41.58)	2.40 (0.00–7.69)
		T/T	63.16 (53.27–72.44)	32.72 (22.87–42.43)	2.14 (0.00–5.50)
		*p^KW^*	0.206	0.318	0.730

*^KW^* Kruskal–Wallis test; *^U^* Mann–Whitney’s U test; * Statistical significance (*p* < 0.05). Showing median and interquartile ranges (p25–p75).

## Data Availability

The original contributions presented in this study are included in the article/Supplementary Material. Further inquiries can be directed to the corresponding authors.

## Supplementary Materials

The following supporting information can be downloaded at: https://www.mdpi.com/article/10.3390/ph18091335/s1, Table S1. Allelic and genotypic frequencies and activity score distribution by *CYP2C8, CYP2C9*, and *CYP2C19* within a sample of Ecuadorian T2DM patients (n = 297). Table S2. SNV allelic and genotypic frequencies in *SLC22A1*, *SLC22A2*, *SLC22A3*, and *ABCB1* (n = 297). Table S3. Correlation between ancestry proportion and allelic frequency in *CYP2C8* variants. Table S4. Correlation between ancestry proportion and allelic frequency in *CYP2C9* variants Table S5. Correlation between ancestry proportion and allelic frequency in *CYP2C19* variants Table S6. Correlation between ancestry proportion and allelic frequency in SNVs in *SLC22A1* and *SLC22A3.* Table S7. Analysis of allelic variants with their respective Taqman PCR-RT assays, enzymatic activity.

## Abbreviations

The following abbreviations are used in this manuscript:

ABCB1	ATP Binding Cassette Subfamily B Member 1
ADRs	Adverse drug reactions
AFR	African
AIMs	Ancestry informative markers
CPIC	Clinical Pharmacogenetics Implementation Consortium Guidelines
CYP450	Cytochrome P450
T2DM	Type 2 diabetes mellitus
EUR	European
IM	Intermediate metabolizer
NATAM	Native American
NM	Normal metabolizer
OCT	Organic cation transporters
PM	Poor metabolizer
P-gp	P-glycoprotein
SNV	Single nucleotide allelic variant
SU	Sulfonylureas
TZD	Thiazolidinediones
