# Supplementary material for: Pharmacogenetics and Molecular Ancestry of SLC22A1, SLC22A2, SLC22A3, ABCB1, CYP2C8, CYP2C9, and CYP2C19 in Ecuadorian Subjects with Type 2 Diabetes Mellitus
Source: Pharmaceuticals (Basel). 2025 Sep 5;18(9):1335. doi: 10.3390/ph18091335 (PMC12472588; doi:10.3390/ph18091335)
Supplement: Supplementary file 1 [file pharmaceuticals-18-01335-s001.zip › pharmaceuticals-3834233-supplementary/Table _S3.pdf]

Table S3. Correlation between ancestry proportion and allelic frequency in *CYP2C8* variants.

	Allelic variant of *CYP2C8*		
	Native-American ancestry		
	wt	**3*	**4*
Rho ^{s}	0.193	-0.140	-0.137
p^{s}	<0.001*	0.015*	0.018*
	European ancestry		
Rho ^{s}	-0.170	0.112	0.142
p^{s}	0.003*	0.053	0.014*
	African ancestry		
Rho ^{s}	-0.093	0.118	0.023
p^{s}	0.108	0.091	0.693

Rho^{s}, Spearman's correlation coefficient; p^{s}, p value for Spearman's correlation test; *Statistical significance (p<0.05).
