# Supplementary material for: Pharmacogenetics and Molecular Ancestry of SLC22A1, SLC22A2, SLC22A3, ABCB1, CYP2C8, CYP2C9, and CYP2C19 in Ecuadorian Subjects with Type 2 Diabetes Mellitus
Source: Pharmaceuticals (Basel). 2025 Sep 5;18(9):1335. doi: 10.3390/ph18091335 (PMC12472588; doi:10.3390/ph18091335)
Supplement: Supplementary file 1 [file pharmaceuticals-18-01335-s001.zip › pharmaceuticals-3834233-supplementary/Table_S1.pdf]

Table S1. Allelic and genotypic frequencies and activity score distribution by *CYP2C8*, *CYP2C9*, and *CYP2C19* within a sample of Ecuadorian T2DM patients (n= 297).

	Genotypic frequency		Allele Frequency		Activity score	
	n= 297					
**Gen**		n (%)		(%)		n (%)
CYP2C8	*1/*1	257 (86.53)	*1	93.26	1	0 (0.00)
	*1/*3	31 (10.43)	*3	5.21	1.5	40 (13.46)
	*1/*4	9 (3.03)	*4	1.51	2	257 (86.53)
	*3/*4	0 (0.00)				
rs11572080	GG	266 (89.56)	G	94.78		
	GA	31 (10.43)	A	5.22		
	AA	0 (0.00)	*p* ǂ	0.342		
rs1058930	CC	0 (0.00%)	C	98.48		
	CG	9 (3.03)	G	1.52		
	GG	288 (96.69)	*p* ǂ	0.790		
CYP2C9[a]	*1/*1	252 (85.13)	*1	92.39	0.5	0 (0.00)
	*1/*2	29 (9.79)	*2	5.23	1	15 (5.06)
	*1/*3	14 (4.72)	*3	2.36	1.5	29 (9.79)
	*2/*2	1 (0.33)			2	252 (85.13)
	*2/*3	0 (0.00)				
rs1799853	CC	266 (89.86)	C	94.76		
	CT	29 (9.79)	T	5.24		
	TT	1 (0.33)	*p* ǂ	0.825		
rs1057910	AA	282 (95.27)	A	97.64		
	AC	14 (4.72)	C	2.36		
	CC	0 (0.00)	*p* ǂ	0.676		
CYP2C19 [a]	*1/*1	199 (67.22)	*1	82.60	0 PM	4 (1.35)
	*1/*2	57 (19.25)	*2	11.14	1	57 (19.25)
	*1/*4	0 (0.00)	*4	0.00	1.5	1 (0.33)
	*1/*17	34 (11.48)	*17	6.25	2	199 (67.22)
	*2/*2	4 (1.35)			>2 UM	35 (11.82)
	*17/*17	1 (0.33)				
	*2/*17	1 (0.33)				
rs4244285	GG	234 (79.05)	G	88.85		
	GA	58 (19.59)	A	11.15		
	AA	4 (1.35)	*p* ǂ	0.850		
rs12248560	CC	260 (87.83)	C	93.75		
	CT	35 (11.82)	T	6.25		
	TT	1 (0.33)	*p* ǂ	0.876		

ǂ p value for Pearson's Chi-square test determining Hardy-Weinberg equilibrium. [a]missing (n=1)
