# Supplementary material for: Pharmacogenetics and Molecular Ancestry of SLC22A1, SLC22A2, SLC22A3, ABCB1, CYP2C8, CYP2C9, and CYP2C19 in Ecuadorian Subjects with Type 2 Diabetes Mellitus
Source: Pharmaceuticals (Basel). 2025 Sep 5;18(9):1335. doi: 10.3390/ph18091335 (PMC12472588; doi:10.3390/ph18091335)
Supplement: Supplementary file 1 [file pharmaceuticals-18-01335-s001.zip › pharmaceuticals-3834233-supplementary/Table_S2.pdf]

Table 2. SNV allelic and genotypic frequencies in *SLC22A1*, *SLC22A2*, *SLC22A3*, and *ABCB1* (n= 297 Ecuadorian T2DM patients).

Gene	ID	Genotype	n (%)	Allele Frequency (%)		p value
SLC22A1	rs72552763	GAT/GAT	143 (48.14)	GAT	68.86	0.553
		GAT/del	123 (41.41)	del	31.14	
		del/del	31 (10.43)			
	rs622342	A/A	101 (34.00)	A	58.42	0.932
		A/C	145 (48.82)	C	41.58	
		C/C	51 (17.17)			
	rs12208357	CC	292 (98.31)	C	99.16	0.883
		CT	5 (1.68)	T	0.84	
		TT	0 (0.00)			
	rs2282143[a]	CC	284 (95.94)	C	97.97	0.721
		CT	12 (4.05)	T	2.03	
		TT	0 (0.00)			
	rs594709	AA	195 (65.65)	A	82.15	**0.030***
		AG	98 (32.99)	G	17.85	
		GG	4 (1.34)			
	rs683369	CC	247 (83.16)	C	91.08	0.650
		CG	47 (15.82)	G	8.92	
		GG	3 (1.01)			
	rs628031	GG	213 (71.71)	G	85.02	0.447
		GA	79 (26.59)	A	14.98	
		AA	5 (1.68)			
SLC22A2	rs316019	C/C	277 (93.26)	C	96.46	0.284
		C/A	19 (6.39)	A	3.54	
		A/A	1 (0.33)			
SLC22A3	rs2076828	C/C	224 (75.42)	C	86.53	0.422
		C/G	66 (22.22)	G	13.47	
		G/G	7 (2.35)			
	rs8187725	CC	297 (100)	C	100	-
		CT	0 (0.00)	T	0	
		TT	0 (0.00)			
ABCB1	rs1128503	C/C	70 (23.56)	C	46.30	0.138
		C/T	135 (45.45)	T	53.70	
		T/T	92 (30.97)			
	rs2032582[a]	G/G	71 (28.16)	G	47.63	0.702
		G/A	12 (7.75)	A	4.72	
		A/A	0 (0.00)	T	0.4408	
		G/T	128 (42.85)			
		T/T	69 (15.91)			
		T/A	16 (4.89)			
	rs1045642	C/C	75 (25.25)	C	49.83	0.771
		C/T	146 (49.15)	T	50.17	
		T/T	76 (25.58)			

ǂ p value for Pearson's Chi-square test for Hardy Weinberg Equilibrium;; [a] Undetermined/missing (n=1).
