# Supplementary material for: Pharmacogenetics and Molecular Ancestry of SLC22A1, SLC22A2, SLC22A3, ABCB1, CYP2C8, CYP2C9, and CYP2C19 in Ecuadorian Subjects with Type 2 Diabetes Mellitus
Source: Pharmaceuticals (Basel). 2025 Sep 5;18(9):1335. doi: 10.3390/ph18091335 (PMC12472588; doi:10.3390/ph18091335)
Supplement: Supplementary file 1 [file pharmaceuticals-18-01335-s001.zip › pharmaceuticals-3834233-supplementary/Table_S4.pdf]

Table S4. Correlation between ancestry proportion and allelic frequency in *CYP2C9* variants.

	Allele variant of CYP2C9			
	Native-American ancestry			
	wt	*2	*3	Activity score
Rho ^{s}	0.163	-0.141	-0.070	0.161
p^{s}	0.005*	0.015*	0.227	0.005*
	European ancestry			
Rho ^{s}	-0.127	0.121	0.040	-0.124
p^{s}	0.028*	0.038*	0.488	0.032*
	African ancestry			
Rho ^{s}	-0.130	0.79	0.106	-0.135
p^{s}	0.025*	0.172*	0.069	0.022*

Rho^{s}, Spearman's correlation coefficient; p^{s}, p value for Spearman's correlation test; *Statistical significance($p<0.05$).
