# Supplementary material for: Pharmacogenetics and Molecular Ancestry of SLC22A1, SLC22A2, SLC22A3, ABCB1, CYP2C8, CYP2C9, and CYP2C19 in Ecuadorian Subjects with Type 2 Diabetes Mellitus
Source: Pharmaceuticals (Basel). 2025 Sep 5;18(9):1335. doi: 10.3390/ph18091335 (PMC12472588; doi:10.3390/ph18091335)
Supplement: Supplementary file 1 [file pharmaceuticals-18-01335-s001.zip › pharmaceuticals-3834233-supplementary/Table_S5.pdf]

Table S5. Correlation between ancestry proportion and allelic frequency in *CYP2C19* variants.

	Allele variant of *CYP2C19*				
	Native-American ancestry				
	wt	*2	*4	*17	Activity score
Rho ^{s}	0.087	0.007	-	-0.146	-0.079
p ^{s}	0.133	0.892	-	0.012*	0.175
	European ancestry				
Rho ^{s}	-0.062	0.009	-	0.089	0.035
p ^{s}	0.287	0.868	-	0.125	0.547
	African ancestry				
Rho ^{s}	-0.063	-0.068	-	0.174	0.145
p ^{s}	0.278	0.241	-	0.002*	0.012*

Rho^{s}, Spearman's correlation coefficient; p^{s}, p value for Spearman's correlation test; *Statistical significance($p<0.05$)
