# Supplementary material for: Pharmacogenetics and Molecular Ancestry of SLC22A1, SLC22A2, SLC22A3, ABCB1, CYP2C8, CYP2C9, and CYP2C19 in Ecuadorian Subjects with Type 2 Diabetes Mellitus
Source: Pharmaceuticals (Basel). 2025 Sep 5;18(9):1335. doi: 10.3390/ph18091335 (PMC12472588; doi:10.3390/ph18091335)
Supplement: Supplementary file 1 [file pharmaceuticals-18-01335-s001.zip › pharmaceuticals-3834233-supplementary/Table_S6.pdf]

Table S6. Correlation between ancestry proportion and allelic frequency in SNVs in *SLC22A1* and *SLC22A3*.

		SLC22A1		*SLC22A3*
	rs72552763	rs594709	rs628031	rs2076828
	GAT	A	G	C
		Native-American ancestry		
Rho^s	-0.157	0.147	0.143	0.261
p^s	0.006*	0.011*	0.013*	<0.001*
		European ancestry		
Rho^s	0.162	-0.169	-0.148	-0.230
p^s	0.005*	0.003*	0.010*	<0.001*
		African ancestry		
Rho^s	0.026	0.013	-0.032	-0.127
p^s	0.649	0.812	0.579	0.029*

Rho^s, Spearman's correlation coefficient; p^s, p value for Spearman's correlation test; *Statistical significance($p<0.05$)
