# Supplementary material for: Pharmacogenetics and Molecular Ancestry of SLC22A1, SLC22A2, SLC22A3, ABCB1, CYP2C8, CYP2C9, and CYP2C19 in Ecuadorian Subjects with Type 2 Diabetes Mellitus
Source: Pharmaceuticals (Basel). 2025 Sep 5;18(9):1335. doi: 10.3390/ph18091335 (PMC12472588; doi:10.3390/ph18091335)
Supplement: Supplementary file 1 [file pharmaceuticals-18-01335-s001.zip › pharmaceuticals-3834233-supplementary/Table_S7.pdf]

Table S7. Analysis of allelic variants with their respective Taqman PCR-RT assays, enzymatic activity assignment, and activity score in T2DM Ecuadorian patients (n= 297).

Gen change	rs ID	Nucleotide	Activity	Taqman Assay ID
SLC22A1	rs12208357	c.181C>T		C_30634096_10
	rs2282143	c.1022C>T		C_15877554_40
	rs594709	c.839+597G>A		C___1898206_10
	rs622342	c.1386-2964C>A		C____928527_20
	rs628031	c.1222A>C		C___8709275_60
	rs683369	c.480G>T		C____928536_30
	rs72552763	c.1260_1262del		C_34211613_10
SLC22A2	rs316019	c.808T>G		C___3111809_20
SLC22A3	rs2076828	*698C>G		C___2763995_1_
	rs8187725	c.806C>A		C_30633894_10
ABCB1	rs1128503	c.1236C>T		C_7586662_10
	rs2032582	2677G>T/A		C_11711720C_30<br>C_11711720D_40
	rs1045642	c.3435T>A		C_7586657_20
CYP2C8				
*3	rs11572080	c.2130G>A	*Decreased / 0.5*	C_25625794_10
*4	rs1058930	c.11041C>G	*Decreased / 0.5*	C_25761568_20
CYP2C9				
*2	rs1799853	c.430C>T	*Decreased / 0.5*	C_25625805_10
*3	rs1057910	c.1075A>C	*None / 0*	C_27104892_10
*6	rs9332131	c.818delA	*None / 0*	C_32287221_20
CYP2C19				
*2	rs4244285	c.19154G>A	*None / 0*	C_25986767_70
*3	rs4986893	c.17948 G>A	*None / 0*	C_27861809_10
*4	rs28399504	c.1A>G	*None / 0*	C_30634136_10
*5	rs56337013	c.1297C>T	*None / 0*	C_27861810_10
*17	rs12248560	c.-806C>T	*Increased / 1.5*	C_ _469857_10
